# Oral health knowledge, attitudes, practices and behavioral factors associated with clinical oral health indicators in pregnant women: a cross-sectional study in Spain

**DOI:** 10.3389/froh.2026.1928373

**Published:** 2026-10-01

**Authors:** Francisco Manuel Suárez-Sánchez, Beatriz Prieto-Regueiro, Jesús Miguel Ticona-Flores, Mariangela Tabone, Montserrat Diéguez-Pérez

**Affiliations:** Department of Dentistry, Faculty of Biomedical and Health Sciences, Universidad Europea de Madrid, Campus de Villaviciosa, Madrid, Spain

**Keywords:** Pregnancy, oral health, knowledge, attitudes, practices, oral hygiene, maternal oral health, CAPSOM

## Abstract

**Background:**

Oral health during pregnancy is relevant to maternal well-being and has been associated with perinatal and child oral health outcomes. This study examined associations between a CAPSOM-derived composite knowledge, attitudes, and practices (KAP) score, oral hygiene behaviors, and clinical oral health indicators in pregnant women attending primary health care centers in Lanzarote, Spain.

**Methods:**

An observational cross-sectional study included 293 pregnant women. Sociodemographic, pregnancy-related, anthropometric, behavioral, and smoking-related variables were collected. Thirteen items based on the Maternal Oral Health Knowledge, Attitudes and Practices Questionnaire (CAPSOM) were combined into a composite KAP score. Clinical outcomes included Plaque Index, Gingival Index, and Tricolor Index. Hierarchical multiple linear regression models were fitted, with sensitivity analyses addressing potential overlap between behavioral variables and the composite KAP score. Additional adjusted models examined night-time toothbrushing.

**Results:**

Mean age was 29.81 ± 6.09 years and mean toothbrushing frequency was 2.29 ± 0.98 times/day. Overall, 96.6% brushed at least once daily and 78.5% reported night-time toothbrushing. Final models explained 60.7% of Plaque Index, 53.1% of Gingival Index, and 69.2% of Tricolor Index variance. Toothbrushing frequency was independently associated with lower values for all three outcomes. The composite KAP score showed modest independent associations with Plaque Index (B = −0.006, *Δ*R^2^ = 0.006, *p* = 0.041) and Tricolor Index (B = −0.015, *Δ*R^2^ = 0.021, *p* < 0.001), but not Gingival Index (*p* = 0.401). In the six-item nonbehavioral sensitivity analysis, the association remained significant only for Tricolor Index (B = −0.034, *p* = 0.002). Night-time toothbrushing was independently associated with lower Plaque Index (B = −0.172, *p* < 0.001), Gingival Index (B = −0.132, *p* = 0.015), and Tricolor Index (B = −0.118, *p* = 0.036).

**Conclusions:**

Oral hygiene behaviors, particularly toothbrushing frequency, showed stronger associations with clinical oral health indicators than the composite KAP score. The contribution of the composite score was modest and outcome dependent. These findings support combining oral health education with practical oral hygiene counseling in prenatal care.

## 1 Introduction

Oral health during pregnancy has gained increasing attention in obstetric, dental, and public health research. Although pregnancy is a physiological condition, hormonal, immunological, and metabolic changes may increase susceptibility to oral conditions such as dental caries, gingivitis, and periodontitis (1, 2). Maternal oral health, particularly periodontal status, has also been associated with obstetric outcomes such as preterm birth, low birth weight, and breastfeeding-related factors, highlighting the relevance of oral health within prenatal care and for the well-being of the mother–child dyad (1, 3). Despite this evidence, important gaps persist in pregnant women's oral health-related knowledge, attitudes, and practices. Studies conducted in different geographical contexts have shown that, although many pregnant women report positive attitudes toward oral hygiene, their knowledge and practical skills are often insufficient (3–5). Previous research has described limited awareness regarding the relationship between periodontal disease and pregnancy outcomes, the infectious nature of dental caries, and the importance of preventive dental care during pregnancy (3, 4, 6). These gaps may affect not only maternal oral health but also children's future oral health, as maternal behaviors, preventive practices, and oral health awareness are closely related to the risk of early childhood caries and the transmission of cariogenic bacteria (7–10). Misconceptions about dental care during pregnancy remain an additional barrier. Some pregnant women may avoid dental treatment because of perceived risks to the fetus or uncertainty about the safety of dental procedures during pregnancy (6, 11). In Spain, previous studies have also suggested that some health care professionals, including midwives and midwifery students, may have insufficient or inconsistent knowledge regarding oral health care during pregnancy (12, 13). This lack of consistent oral health guidance may contribute to low dental attendance during pregnancy, as reported in previous literature on determinants of dental care use in this population (11). In this context, improving oral health-related knowledge, attitudes, and practices represents an important preventive strategy for both maternal and child oral health (9, 10). Validated instruments are needed to assess these dimensions in a structured and reproducible way. The Maternal Oral Health Knowledge, Attitudes and Practices Questionnaire (CAPSOM) was developed and validated to evaluate oral health-related knowledge, attitudes, and practices during pregnancy (14). However, most previous studies have focused mainly on self-reported knowledge, attitudes, practices, or general oral health awareness, whereas fewer have examined whether these factors are associated with objective clinical oral health indicators in pregnant women (4, 15, 16). In Spain, evidence directly linking maternal oral health-related knowledge, attitudes, and practices with clinical measures such as plaque accumulation, gingival inflammation, and biofilm maturity remains limited. Therefore, the aim of the present study was to examine the association between CAPSOM-derived oral health knowledge, attitudes, and practices scores, oral hygiene behaviors, and clinical oral health indicators in pregnant women attending primary health care centers in Lanzarote, Spain. We hypothesized that higher CAPSOM-derived scores and more favorable oral hygiene behaviors would be associated with better clinical oral health outcomes.

## 2 Materials and methods

### 2.1 Study design and setting

An observational cross-sectional study was conducted in the Lanzarote Health Area, Spain. The study was approved by the Research Ethics Committee for Medicinal Products of Las Palmas de Gran Canaria (CEIm; approval code: 2023-206-1). All procedures were performed in accordance with the principles of the Declaration of Helsinki, and written informed consent was obtained from all participants before data collection. The study was carried out within the Primary Health Care (PHC) network of the Lanzarote Health Area, which covers the entire island and is organized into seven Basic Health Zones (BHZs), corresponding to the municipalities of Arrecife, San Bartolomé, Tinajo, Tías, Yaiza, Teguise, and Haría. Each BHZ includes one or more public health centers and, where applicable, local health care facilities that provide services to the resident population. Among the seven municipalities of Lanzarote, Arrecife was randomly selected as the recruitment area. To do this, each municipality was assigned a reference number, and one municipality was selected through a random allocation procedure. Participant recruitment took place at the Titerroy and Valterra public health centers within the municipality of Arrecife.

### 2.2 Study population, eligibility criteria, and sample size

The study population consisted of pregnant women residing in Lanzarote who attended routine prenatal care appointments at PHC centers in Arrecife during 2023. Eligible participants were women aged 16 years or older, between 6 and 41 weeks of gestation, holders of a valid Canary Islands Health Card, and willing to participate by providing written informed consent. Women who were unable or unwilling to complete the questionnaire or the clinical oral examination were excluded from the study. According to official records, 1,063 births were registered in the Lanzarote Health Area between January 1 and December 31, 2021. The required sample size was calculated using the standard formula for estimating a proportion in a finite population:

n=**[N** **×** **Z^2^** **×** **p** **×**  **(1−p)]/[d^2^** **×** **(N−1)** **+** **Z^2^** **×** **p** **×** **(1−p)]**

where N is the population size, Z corresponds to the 95% confidence level (Z = 1.96), *p* is the expected proportion, and d is the margin of error. Since no prior prevalence estimate was assumed, *p* was set at 0.5 to obtain the most conservative sample size estimate, and d was set at 0.05. Based on these parameters, the minimum required sample size was 283 participants. A total of 293 pregnant women were finally included in the study, exceeding the minimum required sample size. This calculation was intended to ensure adequate precision for descriptive estimates; therefore, multivariable analyses should be interpreted as explanatory rather than confirmatory.

### 2.3 Recruitment procedure and data collection

During the data collection period, pregnant women attending routine prenatal appointments with midwives at the selected health centers were screened for eligibility. Eligible women were invited to participate during their health center visit, and recruitment was conducted in the waiting areas as part of the routine clinical workflow. Due to the observational design and operational constraints within the health care setting, the number of eligible women who declined participation was not systematically recorded; therefore, a formal refusal rate could not be calculated. Women who agreed to participate received detailed information about the study objectives and procedures, and written informed consent was obtained before data collection. A structured face-to-face interview lasting approximately 10 min was conducted to collect information on pregnancy-related characteristics, sociodemographic variables, anthropometric parameters, oral hygiene behaviors, and smoking habits. Participants subsequently completed 13 items based on the Maternal Oral Health Knowledge, Attitudes and Practices Questionnaire (CAPSOM) framework, designed to assess oral health-related knowledge, attitudes, and practices during pregnancy. Clinical examinations were scheduled two days per week. Participants were instructed to attend the appointment without eating, smoking, chewing gum, or performing oral hygiene procedures for at least one hour before the examination. All clinical assessments were performed between 08:00 and 10:00 a.m. Unstimulated saliva samples were collected first, followed by a comprehensive oral examination. Participants were seated in a dental chair, and examinations were conducted using a No. 5 dental mirror and a World Health Organization (WHO) periodontal probe. The average duration of the clinical examination was approximately 15 min per participant. All clinical oral examinations were performed by the same examiner, who had been previously trained and calibrated. Intra-examiner reliability for the continuous clinical oral health indices was assessed through duplicate examinations in the first 30 consecutive participants enrolled, with an interval of less than seven days between assessments. Reliability was evaluated using a two-way mixed-effects intraclass correlation coefficient (ICC) model for absolute agreement. Excellent intra-examiner reliability was observed for the Gingival Index (ICC = 0.88, 95% CI: 0.76–0.94), Plaque Index (ICC = 0.91, 95% CI: 0.82–0.96), and Tricolor Index (ICC = 0.96, 95% CI: 0.92–0.99).

### 2.4 Study variables and measurements

Pregnancy-related and sociodemographic variables included maternal age, gestational age in weeks, number of pregnancies, socioeconomic status, and educational level. Socioeconomic status was assessed according to the Domingo-Salvany et al. social class classification (17), based on the occupation of the main income provider in the household. The occupational classification criteria are presented in Supplementary Table S1. Armed forces workers were coded as category VI, and inactive individuals were coded as category VII. Educational level was recorded separately according to the categories presented in Supplementary Table S2. Anthropometric variables included body weight, height, and body mass index (BMI). Weight was measured in kilograms, height in centimeters, and BMI was calculated as weight in kilograms divided by height in meters squared. Oral health-related knowledge, attitudes, and practices were assessed using 13 items based on the Maternal Oral Health Knowledge, Attitudes and Practices Questionnaire (CAPSOM) framework described by Ramírez-Trujillo et al. (14). The administered items addressed beliefs, perceptions, and behaviors related to maternal oral health during pregnancy. The full list of items administered in the present study is provided in Supplementary Table S3. For the purposes of the present analyses, responses to the 13 items were combined into a CAPSOM-derived composite knowledge, attitudes, and practices (KAP) score. This composite score was not considered a direct measure of oral health literacy or a newly validated psychometric scale. Items 1–4 were dichotomous and were scored as 1 point for a correct response and 0 points for an incorrect response. Items 5–13 were scored from 0 to 4 according to their Likert response categories. No additional reverse coding was applied. Item scores were summed to obtain the composite score, yielding a theoretical range from 0 to 40. Oral health behavioral variables included smoking habits and oral hygiene practices. Smoking was recorded as the number of cigarettes smoked per day. Toothbrushing frequency was recorded as the number of brushing episodes per day and coded as 0, 1, 2, 3, or 4 times/day. Night-time toothbrushing was recorded separately as a binary variable (0 = no, 1 = yes). Unstimulated salivary flow was measured using the passive drooling technique. Saliva was collected over a 5-minute period, during which participants were instructed to keep their head slightly tilted forward and to avoid oral movements. Salivary flow rate was expressed in mL/min. Clinical oral health variables included plaque accumulation, gingival inflammation, and biofilm maturity. Dental plaque accumulation was assessed using the simplified Silness and Löe Plaque Index (PI). The index teeth examined were 16, 21, 24, 44, 41, and 36, corresponding to a partial selection based on the Ramfjord teeth. Four surfaces of each tooth were examined in the following order: distal, buccal, mesial, and lingual/palatal. When an index tooth was missing, the homologous tooth was examined. Plaque was scored from 0 to 3, where 0 indicated no plaque in the gingival area; 1 indicated plaque not visible to the naked eye but detectable with a probe at the gingival third of the tooth; 2 indicated moderate visible plaque accumulation in the gingival area; and 3 indicated abundant plaque accumulation in the same area, extending toward the adjacent tooth. Gingival inflammation was evaluated using the simplified Löe and Silness Gingival Index (GI) on the same index teeth and surfaces. Gingival status was scored from 0 to 3, where 0 indicated normal gingiva; 1 indicated mild inflammation without bleeding on probing; 2 indicated moderate inflammation characterized by redness, edema, and bleeding on probing; and 3 indicated severe inflammation with pronounced redness and edema, spontaneous bleeding, or ulceration. For each participant, the Plaque Index and Gingival Index were calculated by summing the corresponding scores recorded across all examined surfaces and dividing the resulting sum by the total number of surfaces examined (24 surfaces when all six index teeth were available). Biofilm maturity was assessed using Tri Plaque ID Gel® on the same index teeth and surfaces. The disclosing agent was applied to the dental surfaces using a cotton applicator, after which participants were instructed to rinse gently once and expel the remaining product. Each examined surface was scored according to the disclosed color as follows: 0 = no visible biofilm; 1 = pink, indicating recently formed bacterial plaque; 2 = dark blue, indicating mature bacterial plaque; and 3 = light blue, indicating acid-producing plaque. For each participant, the Tricolor Index was calculated by summing the scores obtained across all examined dental surfaces and dividing the resulting sum by the total number of surfaces examined. Higher Tricolor Index values therefore indicated greater biofilm maturity and/or acidogenic plaque burden.

### 2.5 Statistical analysis

Statistical analyses were performed using IBM SPSS Statistics version 26.0 (IBM Corp., Armonk, NY, USA). All tests were two-sided, and a *p*-value < 0.05 was considered statistically significant. Descriptive statistics were used to summarize the characteristics of the study population. Quantitative variables were expressed as mean ± standard deviation (SD), together with ranges or 95% confidence intervals (95% CI), where appropriate. Categorical variables were reported as absolute frequencies and percentages. Toothbrushing frequency was recorded as the number of brushing episodes per day (0–4 times/day) and was analyzed as a quantitative discrete variable. Night-time toothbrushing was coded as a binary variable (0 = no, 1 = yes). Cigarette consumption was analyzed as the number of cigarettes smoked per day. Complete data were available for all 293 participants. To examine the independent associations between the CAPSOM-derived composite knowledge, attitudes, and practices (KAP) score and clinical oral health outcomes, three separate hierarchical multiple linear regression models were fitted, with Plaque Index, Gingival Index, and Tricolor Index as dependent variables. Predictor variables were selected *a priori* based on their theoretical and clinical relevance and entered sequentially in three blocks. Block 1 included maternal age, gestational week, number of pregnancies, educational level, and occupational socioeconomic status. Educational level and socioeconomic status were entered as categorical variables using indicator (dummy) coding, with secondary education, second cycle, and occupational category IVb, respectively, as reference categories. Block 2 included behavioral variables comprising toothbrushing frequency and number of cigarettes smoked per day. Block 3 included the CAPSOM-derived composite KAP score. Model performance was evaluated using the coefficient of determination (R^2^), adjusted R^2^, and the change in explained variance after each block (*Δ*R^2^). The statistical significance of the improvement in model fit between consecutive blocks was evaluated using the F-change test. Regression results were reported as unstandardized regression coefficients (B), standard errors (SE), standardized coefficients (β), 95% confidence intervals (95% CI), and *p*-values. For educational level and socioeconomic status, their overall contribution to each final model was additionally evaluated using omnibus tests. Prior to interpreting the final models, regression assumptions were systematically evaluated. Linearity and homoscedasticity were assessed through residual plot inspection, normality of residuals was assessed using Q-Q plots, independence of errors was evaluated using the Durbin–Watson statistic, and multicollinearity was examined using tolerance values and variance inflation factors (VIF). Two sensitivity analyses were performed to address the potential conceptual overlap between behavioral variables and the 13-item CAPSOM-derived composite KAP score. First, the behavioral block was omitted, and the composite KAP score was entered after the sociodemographic and pregnancy-related covariates included in Block 1. Second, the original 13-item composite score was replaced by a six-item non-behavioral CAPSOM-derived score comprising items 1, 2, 3, 4, 7, and 8. For the dichotomous items (items 1–4), correct responses were assigned 1 point and incorrect responses 0 points, whereas items 7 and 8 retained their original Likert-scale scores (0–4). Item scores were summed to obtain a total score with a theoretical range from 0 to 12. This six-item score was used exclusively as a sensitivity measure and was not considered a newly validated instrument. In addition, clinical oral health outcomes were initially compared according to night-time toothbrushing behavior using independent-samples *t*-tests. Homogeneity of variances was assessed using Levene's test. When the assumption of equal variances was not met, Welch's *t*-test was used. Between-group mean differences were reported with 95% confidence intervals, and effect sizes were expressed using Hedges’ g. The independent association of night-time toothbrushing with each clinical outcome was subsequently examined using additional multivariable linear regression models. Night-time toothbrushing was entered as a binary predictor while adjusting for maternal age, gestational week, number of pregnancies, educational level, occupational socioeconomic status, overall toothbrushing frequency, cigarette consumption, and the CAPSOM-derived composite KAP score. Multicollinearity was reassessed after simultaneous inclusion of overall toothbrushing frequency and night-time toothbrushing.

## 3 Results

### 3.1 Participant characteristics and CAPSOM-derived KAP score

The study included 293 pregnant women. The mean age was 29.81 ± 6.09 years (range: 16–44 years). Mean body weight was 74.56 ± 16.21 kg, mean height was 161.98 ± 11.39 cm, and mean body mass index (BMI) was 28.39 ± 5.73 kg/m^2^. Socioeconomic status, assessed according to the occupation of the main income provider in the household, was most frequently classified as category IVb (35.2%), followed by IVa (17.7%) and V (17.7%). Together, categories IVa, IVb, and V represented 70.6% of the sample. Regarding educational level, 42.0% of participants had completed secondary education, second cycle, 22.5% secondary education, first cycle, 18.4% had a bachelor's/university degree, and 9.9% had primary education. Oral health-related knowledge, attitudes, and self-reported practices were summarized using the 13-item CAPSOM-derived composite KAP score. The mean composite score was 27.48 ± 6.11, with an observed range of 1–38. Twenty-three participants (7.8%) reported smoking. Mean toothbrushing frequency was 2.29 ± 0.98 times/day. Ten participants (3.4%) reported no toothbrushing; 49 (16.7%) brushed once daily; 112 (38.2%) twice daily; 90 (30.7%) three times daily; and 32 (10.9%) four times daily. Overall, 283 participants (96.6%) reported brushing at least once daily. Night-time toothbrushing was reported by 230 participants (78.5%). Participant characteristics are summarized in Table 1.

**Table 1:** Participant characteristics of the study population (*n* = 293).

Variable	n (%) or mean ± SD
Age, years	29.81 ± 6.09
Age range, years	16–44
Body weight, kg	74.56 ± 16.21
Height, cm	161.98 ± 11.39
Body mass index, kg/m^2^	28.39 ± 5.73
CAPSOM-derived composite KAP score	27.48 ± 6.11
Observed KAP score range	1–38
Smoking	23 (7.8)
Toothbrushing frequency, times/day	2.29 ± 0.98
0 times/day	10 (3.4)
1 time/day	49 (16.7)
2 times/day	112 (38.2)
3 times/day	90 (30.7)
4 times/day	32 (10.9)
Night-time toothbrushing	230 (78.5)
Educational level	
No education/cannot read or write	21 (7.2)
Primary education	29 (9.9)
Secondary education, first cycle	66 (22.5)
Secondary education, second cycle	123 (42.0)
Bachelor's degree/university	54 (18.4)
Occupational socioeconomic status	
Category I	11 (3.8)
Category II	16 (5.5)
Category IIIa	32 (10.9)
Category IIIb	7 (2.4)
Category IIIc	5 (1.7)
Category IVa	52 (17.7)
Category IVb	103 (35.2)
Category V	52 (17.7)
Category VI	3 (1.0)
Category VII	12 (4.1)

### 3.2 Clinical oral health indicators

Clinical oral examinations showed a mean unstimulated salivary flow rate of 0.62 ± 0.32 mL/min. The mean Plaque Index was 0.60 ± 0.40, the mean Gingival Index was 0.44 ± 0.38, and the mean Tricolor Index, reflecting biofilm maturity, was 0.92 ± 0.49. Complete descriptive statistics for clinical oral health variables are presented in Table 2.

**Table 2:** Clinical oral health indicators of the study population (*n* = 293).

Variable	Mean	SD	95% CI	Minimum	Maximum
Unstimulated salivary flow, mL/min	0.62	0.32	0.58–0.66	0.02	1.60
Plaque Index	0.60	0.40	0.55–0.65	0.00	2.08
Gingival Index	0.44	0.38	0.40–0.48	0.00	1.71
Tricolor Index	0.92	0.49	0.86–0.98	0.17	2.21

### 3.3 Hierarchical regression models predicting clinical oral health outcomes

The results of the hierarchical multiple linear regression analyses for the three clinical oral health outcomes are presented in Table 3. The sequential contribution of sociodemographic and pregnancy-related variables (Block 1), behavioral variables (Block 2), and the CAPSOM-derived composite KAP score (Block 3) was assessed by examining changes in R^2^ across successive models. For the Plaque Index, sociodemographic and pregnancy-related variables entered in Block 1 explained 35.7% of the variance (R^2^ = 0.357; adjusted R^2^ = 0.320; *p* < 0.001). Adding toothbrushing frequency and cigarette consumption in Block 2 significantly increased the explained variance by 24.3% (*Δ*R^2^ = 0.243; F-change = 83.36; *p* < 0.001). Addition of the CAPSOM-derived composite KAP score in Block 3 produced a further 0.6% increase in explained variance (*Δ*R^2^ = 0.006; F-change = 4.22; *p* = 0.041). The final model explained 60.7% of the variance (R^2^ = 0.607; adjusted R^2^ = 0.579). Higher toothbrushing frequency was independently associated with lower Plaque Index scores (B = −0.209, SE = 0.019, β = −0.518, 95% CI −0.247 to −0.170, *p* < 0.001). The CAPSOM-derived composite KAP score was also inversely associated with Plaque Index, although the magnitude of the association was small (B = −0.006, SE = 0.003, β = −0.099, 95% CI −0.013 to −0.0003, *p* = 0.041). Educational level was a significant overall categorical predictor (*p* < 0.001), whereas socioeconomic status was not (*p* = 0.438). Cigarette consumption was not statistically significant (B = 0.012, *p* = 0.053). For the Gingival Index, Block 1 explained 34.8% of the variance (R^2^ = 0.348; adjusted R^2^ = 0.310; *p* < 0.001). Behavioral variables added 18.2% (*Δ*R^2^ = 0.182; F-change = 52.99; *p* < 0.001), whereas addition of the CAPSOM-derived composite KAP score did not significantly improve model fit (*Δ*R^2^ = 0.001; F-change = 0.71; *p* = 0.401). The final model explained 53.1% of the variance (R^2^ = 0.531; adjusted R^2^ = 0.498). Higher toothbrushing frequency was independently associated with lower Gingival Index scores (B = −0.170, SE = 0.021, β = −0.435, 95% CI −0.210 to −0.129, *p* < 0.001). Gestational week (B = 0.008, SE = 0.002, β = 0.202, 95% CI 0.004 to 0.011, *p* < 0.001) and cigarette consumption (B = 0.020, SE = 0.006, β = 0.140, 95% CI 0.008 to 0.033, *p* = 0.002) were positively associated with Gingival Index. The CAPSOM-derived composite KAP score was not independently associated with Gingival Index (B = −0.003, *p* = 0.401). Educational level was significant overall (*p* < 0.001), whereas socioeconomic status was not (*p* = 0.135). For the Tricolor Index, Block 1 explained 42.7% of the variance (R^2^ = 0.427; adjusted R^2^ = 0.394; *p* < 0.001). Behavioral variables increased the explained variance by 24.4% (*Δ*R^2^ = 0.244; F-change = 101.71; *p* < 0.001), and the CAPSOM-derived composite KAP score contributed a further 2.1% (*Δ*R^2^ = 0.021; F-change = 18.43; *p* < 0.001). The final model explained 69.2% of the variance (R^2^ = 0.692; adjusted R^2^ = 0.671). Higher toothbrushing frequency was independently associated with lower Tricolor Index scores (B = −0.238, SE = 0.021, β = −0.476, 95% CI −0.280 to −0.196, *p* < 0.001). Cigarette consumption was associated with higher scores (B = 0.018, SE = 0.007, β = 0.096, 95% CI 0.005 to 0.031, *p* = 0.007). The CAPSOM-derived composite KAP score remained independently associated with lower Tricolor Index scores (B = −0.015, SE = 0.003, β = −0.182, 95% CI −0.021 to −0.008, *p* < 0.001). Educational level was significant overall (*p* < 0.001), whereas socioeconomic status was not (*p* = 0.110). The complete coefficients from the final multivariable models are presented in Table 4. After adjustment for all covariates, toothbrushing frequency remained independently associated with all three clinical outcomes, whereas the independent contribution of the CAPSOM-derived composite KAP score differed across outcomes. Educational level showed a significant overall association with each clinical outcome, whereas socioeconomic status was not significant in the omnibus tests.

**Table 3:** Hierarchical model fit for clinical oral health outcomes.

Outcome	Block	R^2^	Adjusted R^2^	*Δ*R^2^	F-change	*p*-change
Plaque Index	1	0.357	0.320	0.357	9.60	<0.001
	2	0.601	0.574	0.243	83.36	<0.001
	3	0.607	0.579	0.006	4.22	0.041
Gingival Index	1	0.348	0.310	0.348	9.20	<0.001
	2	0.530	0.499	0.182	52.99	<0.001
	3	0.531	0.498	0.001	0.71	0.401
Tricolor Index	1	0.427	0.394	0.427	12.87	<0.001
	2	0.671	0.650	0.244	101.71	<0.001
	3	0.692	0.671	0.021	18.43	<0.001

Block 1 included maternal age, gestational week, number of pregnancies, educational level, and occupational socioeconomic status. Educational level and socioeconomic status were entered as categorical variables using dummy coding. Block 2 included toothbrushing frequency (times/day) and cigarettes/day. Block 3 included the CAPSOM-derived composite knowledge, attitudes, and practices (KAP) score. R^2^, coefficient of determination; *Δ*R^2^, change in explained variance between consecutive blocks.

**Table 4:** Final multivariable regression models for plaque index, gingival index, and tricolor index (*n* = 293).

	Plaque index			Gingival index			Tricolor index		
	B (SE)	95% CI	*p*	B (SE)	95% CI	*p*	B (SE)	95% CI	*p*
Predictor									
Maternal age	−0.001 (0.003)	−0.007 to 0.005	0.754	0.000 (0.003)	−0.006 to 0.006	0.895	−0.003 (0.003)	−0.010 to 0.003	0.281
Gestational week	0.001 (0.002)	−0.002 to 0.004	0.379	0.008 (0.002)	0.004 to 0.011	<0.001	0.001 (0.002)	−0.002 to 0.004	0.521
Number of pregnancies	0.011 (0.013)	−0.014 to 0.037	0.374	0.003 (0.014)	−0.024 to 0.030	0.826	0.017 (0.014)	−0.010 to 0.045	0.223
Educational level (overall)									
			**<0**.**001**			**<0**.**001**			**<0**.**001**
No education/cannot read or write vs. Secondary education, second cycle	0.237 (0.066)	0.106 to 0.367	<0.001	0.210 (0.070)	0.072 to 0.348	0.003	0.172 (0.073)	0.028 to 0.315	0.019
First grade vs. Secondary education, second cycle	0.187 (0.059)	0.071 to 0.302	0.002	0.180 (0.062)	0.058 to 0.302	0.004	0.228 (0.064)	0.101 to 0.355	<0.001
Second grade, first cycle vs. Secondary education, second cycle	0.156 (0.042)	0.074 to 0.237	<0.001	0.158 (0.044)	0.071 to 0.244	<0.001	0.198 (0.046)	0.108 to 0.287	<0.001
Bachelor's degree/university vs. Secondary education, second cycle	−0.040 (0.050)	−0.139 to 0.059	0.425	−0.022 (0.053)	−0.127 to 0.082	0.674	−0.189 (0.055)	−0.297 to −0.080	0.001
Socioeconomic status (overall)									
			**0**.**438**			**0**.**135**			**0**.**110**
I vs. IVb	−0.182 (0.087)	−0.353 to −0.012	0.036	−0.179 (0.092)	−0.360 to 0.001	0.052	−0.223 (0.095)	−0.410 to −0.036	0.020
II vs. IVb	−0.020 (0.080)	−0.179 to 0.138	0.801	−0.035 (0.085)	−0.202 to 0.133	0.684	0.067 (0.088)	−0.106 to 0.241	0.447
IIIa vs. IVb	−0.044 (0.057)	−0.156 to 0.068	0.440	−0.089 (0.060)	−0.208 to 0.030	0.142	−0.028 (0.063)	−0.151 to 0.095	0.652
IIIb vs. IVb	−0.002 (0.102)	−0.202 to 0.198	0.985	0.081 (0.107)	−0.131 to 0.292	0.454	−0.041 (0.111)	−0.261 to 0.178	0.711
IIIc vs. IVb	−0.058 (0.121)	−0.297 to 0.181	0.632	−0.232 (0.128)	−0.485 to 0.020	0.071	0.057 (0.133)	−0.205 to 0.319	0.667
IVa vs. IVb	−0.048 (0.046)	−0.139 to 0.043	0.303	−0.057 (0.049)	−0.153 to 0.039	0.244	−0.026 (0.051)	−0.126 to 0.074	0.604
V vs. IVb	−0.045 (0.045)	−0.133 to 0.043	0.316	−0.004 (0.048)	−0.097 to 0.090	0.938	−0.041 (0.049)	−0.138 to 0.056	0.412
VI vs. IVb	−0.228 (0.153)	−0.530 to 0.073	0.137	−0.127 (0.162)	−0.446 to 0.192	0.433	−0.217 (0.168)	−0.547 to 0.114	0.198
VII vs. IVb	0.086 (0.079)	−0.070 to 0.242	0.278	0.152 (0.084)	−0.013 to 0.317	0.071	0.167 (0.087)	−0.004 to 0.338	0.056
Toothbrushing frequency, times/day	−0.209 (0.019)	−0.247 to −0.170	<0.001	−0.170 (0.021)	−0.210 to −0.129	<0.001	−0.238 (0.021)	−0.280 to −0.196	<0.001
Cigarettes/day	0.012 (0.006)	<0.001 to 0.024	0.053	0.020 (0.006)	0.008 to 0.033	0.002	0.018 (0.007)	0.005 to 0.031	0.007
CAPSOM-derived composite KAP score	−0.006 (0.003)	−0.013 to −0.0003	0.041	−0.003 (0.003)	−0.009 to 0.004	0.401	−0.015 (0.003)	−0.021 to −0.008	<0.001

B, unstandardized regression coefficient; SE, standard error; CI, confidence interval. Reference categories were Secondary education, second cycle, for educational level and occupational category IVb for socioeconomic status. Overall *p*-values for educational level and socioeconomic status correspond to omnibus tests. All models included 293 participants.

Bold *p*-values indicate omnibus tests for the categorical predictors educational level and socioeconomic status.

### 3.4 Sensitivity analyses

When behavioral variables were omitted and the 13-item CAPSOM-derived composite KAP score was added to the sociodemographic and pregnancy-related covariates, the composite KAP score was significantly associated with all three clinical outcomes. It accounted for an additional 7.5% of the variance in Plaque Index (B = −0.020, β = −0.314, *Δ*R^2^ = 0.075, *p* < 0.001), 4.5% of the variance in Gingival Index (B = −0.015, β = −0.242, *Δ*R^2^ = 0.045, *p* < 0.001), and 11.3% of the variance in Tricolor Index (B = −0.031, β = −0.386, *Δ*R^2^ = 0.113, *p* < 0.001). In a second sensitivity analysis, the original 13-item composite KAP score was replaced by a six-item non-behavioral CAPSOM-derived score comprising items 1, 2, 3, 4, 7, and 8. The six-item score had a mean of 9.49 ± 1.84 and an observed range of 1–12. In the fully adjusted models, the six-item score was not independently associated with Plaque Index (B = −0.017, SE = 0.010, β = −0.081, 95% CI −0.037 to 0.002, *p* = 0.075) or Gingival Index (B = −0.014, SE = 0.010, β = −0.069, 95% CI −0.035 to 0.006, *p* = 0.162). In contrast, it remained independently associated with lower Tricolor Index scores (B = −0.034, SE = 0.011, β = −0.126, 95% CI −0.055 to −0.012, *p* = 0.002), accounting for an additional 1.1% of the variance (*Δ*R^2^ = 0.011). The six-item CAPSOM-derived score was used only as a sensitivity measure and should not be interpreted as a newly validated scale. The results of both sensitivity analyses are summarized in Table 5.

**Table 5:** Sensitivity analyses of CAPSOM-derived scores.

Analysis	Outcome	B	SE	β	95% CI	*p*	R^2^	Adjusted R^2^	*Δ*R^2^
Block 1 + 13-item composite KAP score; behavioral block omitted	Plaque Index	−0.020	0.003	−0.314	−0.027 to −0.014	<0.001	0.433	0.401	0.075
	Gingival Index	−0.015	0.003	−0.242	−0.022 to −0.009	<0.001	0.392	0.358	0.045
	Tricolor Index	−0.031	0.004	−0.386	−0.039 to −0.024	<0.001	0.541	0.515	0.113
Six-item nonbehavioral CAPSOM-derived score; fully adjusted	Plaque Index	−0.017	0.010	−0.081	−0.037 to 0.002	0.075	0.605	0.578	0.005
	Gingival Index	−0.014	0.010	−0.069	−0.035 to 0.006	0.162	0.533	0.501	0.003
	Tricolor Index	−0.034	0.011	−0.126	−0.055 to −0.012	0.002	0.682	0.660	0.011

B, unstandardized regression coefficient; SE, standard error; β, standardized regression coefficient; CI, confidence interval; R^2^, coefficient of determination; *Δ*R^2^, change in explained variance. The first sensitivity analysis omitted the behavioral block and added the 13-item CAPSOM-derived composite KAP score after the sociodemographic and pregnancy-related covariates. The second sensitivity analysis used a six-item nonbehavioral CAPSOM-derived score comprising items 1, 2, 3, 4, 7, and 8 in the fully adjusted models. The six-item score was used only as a sensitivity measure and should not be interpreted as a newly validated scale.

### 3.5 Night-time toothbrushing analyses

In the unadjusted analysis, participants who did not report brushing their teeth at night showed significantly higher Plaque Index, Gingival Index, and Tricolor Index values than those who reported night-time toothbrushing. For the Plaque Index, mean values were 1.058 ± 0.310 among participants who did not brush at night and 0.471 ± 0.316 among those who did. Homogeneity of variances was satisfied (Levene's *p* = 0.193), and the mean difference was 0.587 (95% CI 0.499 to 0.675; *p* < 0.001), with a large effect size (Hedges’ g = 1.859). For the Gingival Index, mean values were 0.828 ± 0.363 and 0.338 ± 0.318, respectively. Homogeneity of variances was also satisfied (Levene's *p* = 0.070), and the mean difference was 0.489 (95% CI 0.397 to 0.581; *p* < 0.001), with a large effect size (Hedges’ g = 1.489). For the Tricolor Index, mean values were 1.476 ± 0.309 among participants who did not brush at night and 0.768 ± 0.417 among those who did. Homogeneity of variances was not satisfied (Levene's *p* < 0.001); therefore, Welch's *t*-test was used. The mean difference was 0.708 (95% CI 0.614 to 0.802; *p* < 0.001), with a large effect size (Hedges’ g = 1.779). Detailed results of the unadjusted comparisons, including variance homogeneity testing, mean differences with 95% confidence intervals, and effect sizes, are presented in Supplementary Table S4, while mean ± standard deviation values are shown in Figure 1. In additional multivariable analyses, night-time toothbrushing remained independently associated with all three clinical oral health outcomes after adjustment for maternal age, gestational week, number of pregnancies, educational level, occupational socioeconomic status, overall toothbrushing frequency, cigarette consumption, and the CAPSOM-derived composite KAP score. Participants reporting night-time toothbrushing had lower adjusted Plaque Index scores than those who did not brush at night (B = −0.172, SE = 0.050, β = −0.179, 95% CI −0.271 to −0.073, *p* < 0.001). Similar associations were observed for Gingival Index (B = −0.132, SE = 0.054, β = −0.141, 95% CI −0.238 to −0.026, *p* = 0.015) and Tricolor Index (B = −0.118, SE = 0.056, β = −0.099, 95% CI −0.228 to −0.008, *p* = 0.036). Overall toothbrushing frequency remained independently associated with each outcome after inclusion of night-time toothbrushing: Plaque Index B = −0.170 (*p* < 0.001), Gingival Index B = −0.140 (*p* < 0.001), and Tricolor Index B = −0.212 (*p* < 0.001). No problematic multicollinearity was observed; the maximum VIF was 2.18, with VIF values of 2.18 for overall toothbrushing frequency and 1.97 for night-time toothbrushing. The adjusted associations are presented in Table 6.

**Figure 1:**
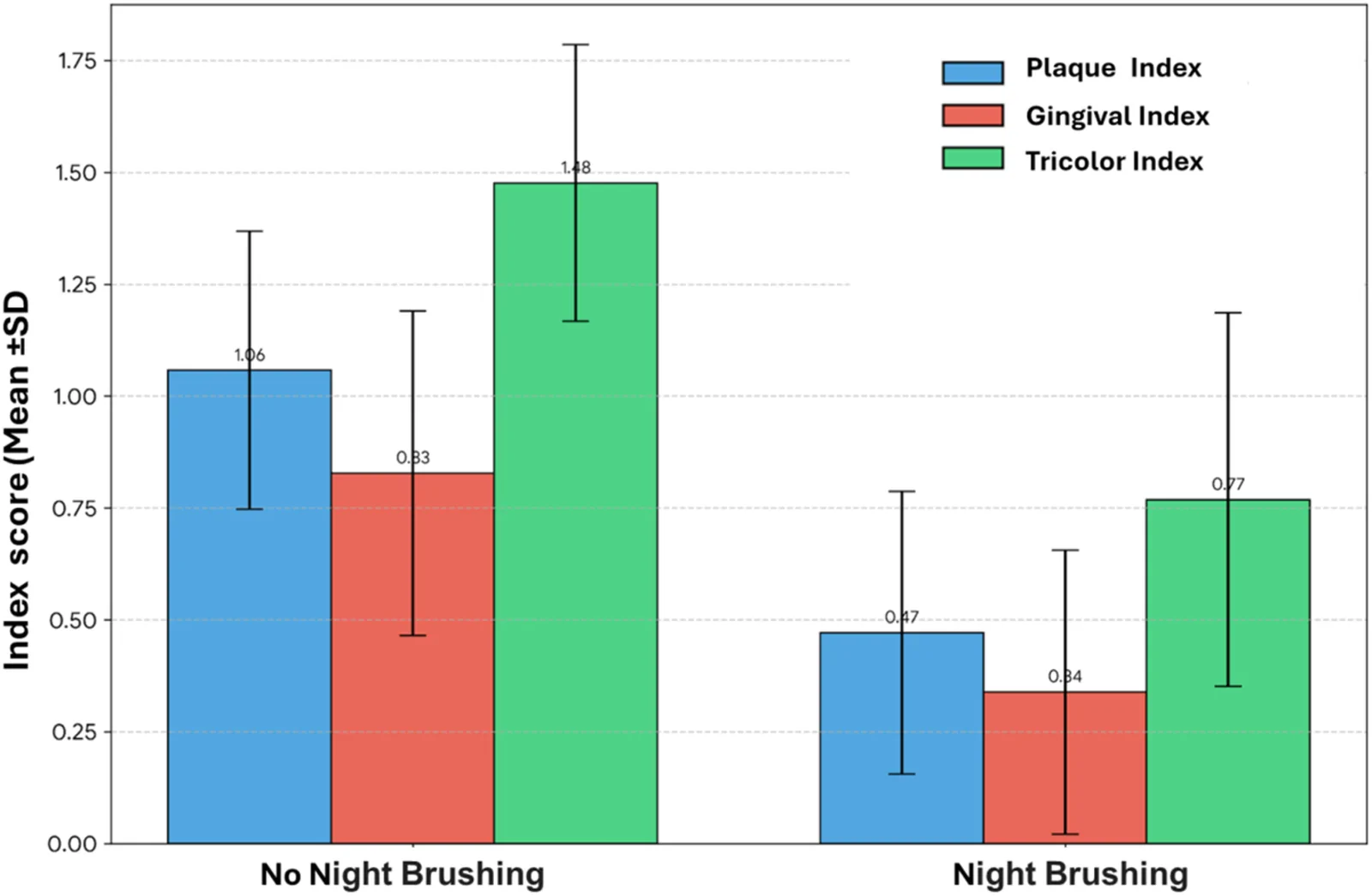
Clinical oral health indicators according to night-time toothbrushing behavior. Bar plots show mean ± standard deviation values for Plaque Index, Gingival Index, and Tricolor Index according to self-reported night-time toothbrushing. Participants who did not report brushing their teeth at night showed higher unadjusted values for all three clinical oral health indicators compared with those who reported night-time toothbrushing. All unadjusted between-group comparisons were statistically significant (*p* < 0.001).

**Table 6:** Adjusted associations of night-time toothbrushing with clinical oral health outcomes.

Outcome	B	SE	β	95% CI	*p*	Model R^2^	Adjusted R^2^
Plaque Index	−0.172	0.050	−0.179	−0.271 to −0.073	<0.001	0.623	0.595
Gingival Index	−0.132	0.054	−0.141	−0.238 to −0.026	0.015	0.541	0.507
Tricolor Index	−0.118	0.056	−0.099	−0.228 to −0.008	0.036	0.697	0.675

B, unstandardized regression coefficient; SE, standard error; β, standardized regression coefficient; CI, confidence interval; R^2^, coefficient of determination. Night-time toothbrushing was coded as 0 = no and 1 = yes, with no as the reference category. Models were adjusted for maternal age, gestational week, number of pregnancies, educational level, occupational socioeconomic status, overall toothbrushing frequency (times/day), cigarette consumption (cigarettes/day), and the CAPSOM-derived composite knowledge, attitudes, and practices (KAP) score.

## 4 Discussion

The present study examined the associations between a CAPSOM-derived composite knowledge, attitudes, and practices (KAP) score, oral hygiene behaviors, and clinical oral health indicators in pregnant women attending primary health care centers in Lanzarote, Spain. The main finding was that toothbrushing frequency was the strongest and most consistent behavioral factor associated with clinical oral health status. Higher toothbrushing frequency was independently associated with lower Plaque Index, Gingival Index, and Tricolor Index values. In contrast, the CAPSOM-derived composite KAP score showed a smaller and outcome-dependent contribution: it was independently associated with lower Plaque Index and Tricolor Index values, but not with Gingival Index after adjustment for sociodemographic, pregnancy-related, and behavioral variables. The magnitude of the independent contribution of the composite KAP score was modest compared with that of toothbrushing frequency. Addition of the KAP score to the fully adjusted models explained an additional 0.6% of the variance in Plaque Index and 2.1% of the variance in Tricolor Index, whereas it did not significantly improve the Gingival Index model. Based on the regression coefficients, a 10-point difference in the composite KAP score would correspond to an estimated difference of approximately 0.06 units in Plaque Index and 0.15 units in Tricolor Index, after adjustment for the remaining covariates. These findings suggest that the independent statistical contribution of the composite score is relatively small once actual oral hygiene behaviors are considered. The sensitivity analyses further clarified this relationship. When the behavioral block was omitted, the 13-item CAPSOM-derived composite KAP score showed stronger associations with all three clinical outcomes, accounting for an additional 7.5% of the variance in Plaque Index, 4.5% in Gingival Index, and 11.3% in Tricolor Index. However, when the original composite was replaced by a six-item nonbehavioral CAPSOM-derived sensitivity score, the association was attenuated and remained statistically significant only for Tricolor Index. This pattern suggests that part of the association observed for the 13-item composite may reflect the behavioral content included in the score itself. Therefore, the associations between the composite KAP score and clinical outcomes should be interpreted cautiously and should not be considered independent evidence of an effect of knowledge or attitudes alone. The strong and consistent association between toothbrushing frequency and all three clinical outcomes emphasizes the importance of daily oral hygiene practices during pregnancy. Although 96.6% of participants reported brushing their teeth at least once per day, considerable variation remained in plaque accumulation, gingival inflammation, and biofilm maturity. This suggests that reporting daily toothbrushing alone may not fully capture brushing effectiveness, frequency, technique, duration, or the use of complementary oral hygiene measures. Previous studies have similarly shown that positive attitudes and knowledge regarding oral health do not always translate into effective preventive practices during pregnancy (2, 5, 18). Night-time toothbrushing provided additional information beyond overall brushing frequency. Women who did not report brushing their teeth at night had substantially higher unadjusted Plaque Index, Gingival Index, and Tricolor Index values than those who did, with large standardized effect sizes for all three comparisons. Importantly, night-time toothbrushing remained independently associated with all three outcomes after adjustment for maternal age, gestational week, number of pregnancies, educational level, occupational socioeconomic status, overall toothbrushing frequency, cigarette consumption, and the CAPSOM-derived composite KAP score. Overall toothbrushing frequency also remained independently associated with each outcome after night-time toothbrushing was included in the models, and no problematic multicollinearity was observed. These findings suggest that the timing of toothbrushing may provide information that is not fully captured by total daily brushing frequency. The differences observed across the three clinical outcomes are also relevant. Plaque accumulation and biofilm maturity are closely related to oral hygiene practices, whereas gingival inflammation may additionally be influenced by pregnancy-related and biological factors. In the present study, gestational week and cigarette consumption were positively associated with Gingival Index values, whereas the CAPSOM-derived composite KAP score was not. Cigarette consumption was also associated with higher Tricolor Index values. These findings are consistent with previous evidence identifying smoking and inadequate oral hygiene as relevant factors associated with periodontal health during pregnancy (19). Educational level showed a significant overall association with each of the three clinical outcomes. Several educational categories differed from the reference category even after adjustment for behavioral and pregnancy-related factors. These findings are consistent with previous studies indicating that educational factors may be related to oral health knowledge, preventive behaviors, and clinical oral health during pregnancy (3–6). However, occupational socioeconomic status was not statistically significant in the omnibus tests for Plaque Index, Gingival Index, or Tricolor Index in the final models. Therefore, the present findings do not support an independent overall association between occupational socioeconomic status and these clinical outcomes after adjustment for the other covariates. Our findings are broadly consistent with previous research showing that oral health knowledge, awareness, and literacy-related factors may be associated with maternal oral health. Zhang et al. reported associations between oral health literacy, knowledge, and periodontal status among pregnant women in Shanghai (15), while studies conducted in India and Iraq have described gaps in oral and periodontal health awareness among pregnant women (20–22). However, the present results indicate that behavioral variables, particularly toothbrushing frequency, had a substantially stronger association with clinical oral health indicators than the CAPSOM-derived composite KAP score. This distinction is important because greater knowledge or more favorable attitudes do not necessarily translate directly into effective preventive behaviors. These findings have implications for prenatal oral health promotion. Previous studies in Spain have suggested that oral health may not always be fully integrated into prenatal counseling and that knowledge regarding dental care during pregnancy may be inconsistent among health care professionals (12, 13). Limited professional guidance may also contribute to insufficient use of dental services during pregnancy, as previously reported (11). Our results support interventions that go beyond the provision of information alone. Prenatal oral health programs may be more effective when educational content is combined with practical behavioral guidance, including adequate brushing frequency, night-time toothbrushing, appropriate brushing technique, complementary oral hygiene practices, and referral for preventive dental care. The CAPSOM-derived composite used in the present study should be interpreted specifically as a measure combining knowledge, attitudes, and self-reported practices rather than as a direct measure of oral health literacy. The administered questionnaire was based on CAPSOM, an instrument developed to assess maternal oral health-related knowledge, attitudes, and practices during pregnancy (14). However, the 13 administered items were combined for the purposes of the present analyses, and the resulting composite was not treated as a newly validated psychometric scale. This distinction is particularly important because some of the items assess behaviors that are also related to the behavioral variables included independently in the regression models. The sensitivity analyses were therefore conducted to evaluate the extent to which this overlap influenced the observed associations. Taken together, the findings indicate that clinical oral health indicators during pregnancy were more strongly associated with oral hygiene behaviors than with the CAPSOM-derived composite KAP score. The independent contribution of the composite score was statistically significant for plaque accumulation and biofilm maturity but modest in magnitude, and it was not significant for gingival inflammation. These results suggest that educational interventions alone may have limited impact if they are not accompanied by strategies that facilitate the translation of knowledge and attitudes into effective daily oral hygiene practices and access to preventive dental care.

## 5 Study limitations

Despite the strengths of this study, several limitations should be considered when interpreting the findings. First, the cross-sectional design precludes establishing causal relationships between the CAPSOM-derived composite knowledge, attitudes, and practices (KAP) score, oral hygiene behaviors, and clinical oral health outcomes. Therefore, the observed associations should be interpreted as correlational rather than causal. Although Arrecife was randomly selected as the recruitment municipality and participants were recruited within primary health care settings, the sample was clinic-based and was not fully representative of the general population of pregnant women. Consequently, the findings may not be generalizable to all pregnant women in Spain or to populations with different sociodemographic, geographic, or health care access profiles. The refusal rate during recruitment was not systematically recorded because of logistical constraints in the clinical setting. Therefore, potential selection bias cannot be excluded. Women who agreed to participate may have been more interested in health or oral health than those who declined participation. This possibility is particularly relevant given that 96.6% of participants reported brushing their teeth at least once per day. Accordingly, favorable oral hygiene practices and KAP-related characteristics may have been overrepresented in the study sample, potentially limiting external validity and reducing variability in some behavioral measures. Several variables, including oral hygiene behaviors and smoking habits, were self-reported and may therefore have been affected by recall or social desirability bias. In particular, self-reported toothbrushing frequency does not provide information on brushing effectiveness, duration, technique, or the quality of interdental cleaning. The CAPSOM-derived composite used in the present analyses should not be interpreted as a direct measure of oral health literacy or as a newly validated psychometric scale. The 13 administered items were combined to obtain a composite measure of knowledge, attitudes, and self-reported practices for the purposes of the present analyses. Moreover, some of these items include self-reported oral hygiene practices that conceptually overlap with behavioral variables entered separately into the regression models, particularly toothbrushing frequency. Although multicollinearity diagnostics did not indicate problematic collinearity, this overlap may have influenced the apparent independent contribution of the composite KAP score. The sensitivity analyses partly addressed this issue by examining models without the behavioral block and by using a six-item nonbehavioral CAPSOM-derived sensitivity score. Nevertheless, the results of the composite score should be interpreted cautiously. Although the regression models were adjusted for relevant sociodemographic, pregnancy-related, and behavioral variables, residual confounding cannot be excluded because of the observational nature of the study. Factors such as dietary habits, previous periodontal history, detailed access to dental care, oral hygiene technique, interdental cleaning, use of fluoride-containing products, systemic inflammation, and pregnancy-related hormonal variability may have influenced the clinical oral health indicators but were not included in the present models. Future longitudinal studies, ideally including follow-up throughout pregnancy and the postpartum period, would be useful to clarify the temporal relationships between oral health knowledge, attitudes, practices, behaviors, and changes in periodontal status. Interventional studies are also needed to determine whether oral health education combined with practical hygiene instruction and facilitated access to preventive dental care can improve clinical oral health outcomes during pregnancy.

## 6 Conclusions

In this sample of pregnant women attending primary health care centers in Lanzarote, toothbrushing frequency was the strongest and most consistent behavioral factor associated with clinical oral health indicators. Higher toothbrushing frequency was independently associated with lower plaque accumulation, lower gingival inflammation, and lower biofilm maturity. CAPSOM-derived composite knowledge, attitudes, and practices (KAP) score showed modest independent associations with Plaque Index and Tricolor Index, but it was not significantly associated with Gingival Index after adjustment for sociodemographic, pregnancy-related, and behavioral variables. Sensitivity analyses further suggested that part of the association observed for the 13-item composite score may reflect the behavioral content included in the score. Night-time toothbrushing was also independently associated with lower Plaque Index, Gingival Index, and Tricolor Index values after adjustment for overall toothbrushing frequency and the remaining covariates. Taken together, these findings indicate that oral hygiene behaviors appear to have a stronger association with clinical oral health during pregnancy than the composite KAP score alone. Prenatal oral health programs should therefore combine oral health education with practical behavioral guidance, including adequate toothbrushing frequency, night-time toothbrushing, appropriate oral hygiene techniques, and facilitated access to preventive dental care.

## Data Availability

The datasets presented in this article are not readily available because the dataset contains anonymized data from human participants and is therefore subject to privacy and ethical restrictions. The data are not publicly available because participants did not provide consent for unrestricted public sharing of the raw dataset. Anonymized data may be made available by the corresponding author upon reasonable request and subject to approval by the relevant ethics committee, where applicable. Requests to access the anonymized dataset may be directed to the corresponding author: Montserrat Diéguez-Pérez, montserrat.dieguez@universidadeuropea.es.

## References

[1] Serrano-Sánchez S González-González J Rodríguez-Martín B Muñoz-Rodríguez V De Las Heras-Corrochano S Criado-Álvarez JJ. Relationship between oral health knowledge and maternal oral health with obstetric risk and breastfeeding. Int J Environ Res Public Health. (2022) 19(13):7797. 10.3390/ijerph19137797 PMC9265655 35805455

[2] Przeklasa-Bierowiec A Jakubik A Szczeklik K Majewska I Marcinek A Pytko-Polończyk J. Awareness of oral health prophylaxis in pregnant women. Folia Med Cracov. (2020) 60(3):99–112. 10.24425/fmc.2020.135799 33582749

[3] Ahmed Javali M Abullais Saquib S Abdul Khader M Khalid I Yahya AlShahrani A Ahmed Kanji M. Oral health knowledge, attitude, and practice of pregnant women in deccan, south India: a cross-sectional prenatal survey. J Med Life. (2022) 15(3):420–4. 10.25122/jml-2019-0095 35449998 PMC9015187

[4] Chawłowska E Karasiewicz M Lipiak A Staszewski R Cofta M Biskupska M. Oral health behaviours, knowledge, and literacy of expectant mothers: a cross-sectional study among maternity ward patients. Int J Environ Res Public Health. (2022) 19(18):11762. 10.3390/ijerph191811762 PMC9517493 36142035

[5] Wassihun B Ayinalem A Beyene K. Oral health knowledge, attitude, and practice of pregnant women attending antenatal care at public hospitals of bench-sheko zone, southwest Ethiopia. BMC Oral Health. (2023) 23(1):33. 10.1186/s12903-023-02737-136670429

[6] Barbosa MCF Da Rocha NB Rodrigues HSG De Oliveira DSB Fernandes LA De Lima DC. Maternal knowledge of oral health during pregnancy and childbirth. Matern Child Health J. (2023) 27(9):1607–15. 10.1007/s10995-023-03697-2 37394617

[7] Liza Z Vabitha S. Infant oral health awareness among expectant mothers in mangalore and influence of sociodemographic factors: a survey. J Indian Soc Pedod Prev Dent. (2025) 43(1):73–9. 10.4103/jisppd.jisppd_32_25 40159608

[8] Domosławska-Żylińska K Łopatek M Krysińska-Pisarek M Wiśniewska P. Polish pregnant women's Knowledge on early childhood caries prevention and oral hygiene in children. BMC Public Health. (2024) 24(1):70. 10.1186/s12889-023-17604-5 PMC10763392 38166976

[9] Xiao J Alkhers N Kopycka-Kedzierawski DT Billings RJ Grier MA Meyerowitz C. Prenatal oral health care and early childhood caries: a preventive strategy. Med Sci Monit. (2020) 26:e924686. 10.12659/MSM.924686

[10] Sampaio JRF Vidal SA De Goes PSA Bandeira PFR Cabral Filho JE. Sociodemographic, behavioral and oral health factors in maternal and child health: an interventional and associative study from the network perspective. Int J Environ Res Public Health. (2021) 18(8):3895. 10.3390/ijerph18083895 33917677 PMC8067955

[11] Rocha JS Arima LY Werlinger AS Karic T Rodriguez-Martinez M Villena N. Determinants of dental care attendance during pregnancy: a systematic review. Caries Res. (2018) 52(1–2):139–52. 10.1159/000481408 29316548

[12] Touriño S Suárez-Cotelo MC Núñez-Iglesias MJ Domínguez-Martís EM Mosteiro-Miguéns DG López-Ares D. Knowledge, attitudes, and practices of Spanish midwives and midwifery students toward oral healthcare during pregnancy. Int J Environ Res Public Health. (2021) 18(11):6089. 10.3390/ijerph18116089 PMC8200230 34200062

[13] Martínez-Beneyto Y Poza Pascual A Carazo C Serna-Muñoz C Pérez-Silva A Ortiz-Ruiz AJ. Conocimientos, práctica y actitudes hacia la salud oral entre matronas de españa. Educ Med. (2024) 25(2):100880. 10.1016/j.edumed.2023.100880

[14] Ramírez-Trujillo MA Villanueva-Vilchis MC Aguilar-Díaz FC de la Fuente-Hernández J Faustino-Silva DD Gaitán-Cepeda LA. Validation of an instrument to determine oral health knowledge, attitudes, and practices during pregnancy. BMC Oral Health. (2021) 21(1):554. 10.1186/s12903-021-01898-1 PMC8557068 34715826

[15] Zhang X Gu H Zhang Q Ow A Zhang J. Oral health status and literacy/knowledge amongst pregnant women in Shanghai. Int Dent J. (2023) 73(1):112–8. 10.1016/j.identj.2022.06.012 PMC10023588 35835596

[16] Melo P Ferreira de Mello A Alves S A cross-sectional study: dental caries and its relationship with oral health literacy among pregnant women. Pesqui Bras Odontopediatria Clin Integr. (2023) 23:e220054. 10.1590/pboci.2023.054

[17] Domingo-Salvany A Bacigalupe A Carrasco JM Espelt A Ferrando J Borrell C. Propuestas de clase social neoweberiana y neomarxista a partir de la clasificación nacional de ocupaciones 2011. Gac Sanit. (2013) 27(3):263–72. 10.1016/j.gaceta.2012.12.009 23394892

[18] Lo Giudice R Martinelli C Alibrandi A Mondo A Venezia R Cannarozzo MG. Multicenter cross-sectional study of oral health and hygiene practices among pregnant women. J Clin Med. (2024) 13(23):7315. 10.3390/jcm13237315 39685772 PMC11642594

[19] Huang YF Chang YC Jeng JH. Risk factors associated with periodontal disease and its impact on quality of life among pregnant women. J Obstet Gynaecol. (2023) 43(1):2264382. 10.1080/01443615.2023.2264382 37830210

[20] Nikki Mustafa S Singh R Pandit S Antra Ash R Oral health status and awareness among pregnant women: a cross-sectional study in meerut, India. J Pharm Bioallied Sci. (2025) 17(Suppl 3):S2779–81. 10.4103/jpbs.jpbs_1168_25 41164607 PMC12564009

[21] Surabhi D Mamatha S. Knowledge, awareness and adverse effect of poor oral health and periodontal status in pregnant women: an evaluative study. Indian J Dent Res. (2025) 36(1):7–11. 10.4103/ijdr.ijdr_715_23 40190109

[22] Mahmood AA Ali BJ Hassan BK. The link between periodontal hygiene status and pregnancy: awareness and understanding among Iraqi pregnant women. Int J Dent. (2025) 2025:8889305. 10.1155/ijod/8889305 40551784 PMC12185210

