# Supplementary material for: Oral health knowledge, attitudes, practices and behavioral factors associated with clinical oral health indicators in pregnant women: a cross-sectional study in Spain
Source: Front Oral Health. 2026 Oct 1;7:1928373. doi: 10.3389/froh.2026.1928373 (PMC13668602; doi:10.3389/froh.2026.1928373)
Supplement: Supplementary file 1 [file Supplementaryfile1.docx]

**Supplementary Table S1. Occupational socioeconomic status classification according to Domingo-Salvany et al. (17).**

| **Category** | **Occupational classification** |
| --- | --- |
| I | Managers in public administration and companies with 10 or more employees; professions associated with second- and third-cycle university degrees. |
| II | Managers of companies with fewer than 10 employees; professions associated with first-cycle university degrees; technicians and support professionals; artists and athletes. |
| IIIa | Administrative employees and professionals supporting administrative and financial management; personal and security service workers. |
| IIIb | Self-employed workers. |
| IIIc | Supervisors of manual workers. |
| IVa | Skilled manual workers. |
| IVb | Semi-skilled manual workers. |
| V | Unskilled workers. |
| VI | Armed forces workers. |
| VII | Inactive population, including non-workers. |

**Supplementary Table S2. Educational level classification.**

| **Category** | **Educational level classification** |
| --- | --- |
| 1 | No education/cannot read or write |
| 2 | Primary education |
| 3 | Secondary education, first cycle |
| 4 | Secondary education, second cycle |
| 5 | University |

**Supplementary Table S3. Questionnaire items administered in the present study and used to derive the CAPSOM-derived composite KAP score.**

| **ITEM** | **Questionnaire item** |
| --- | --- |
| 1 | Tooth decay and bleeding gums can worsen during pregnancy. |
| 2 | Gum problems can affect my pregnancy and cause problems during my baby’s birth. |
| 3 | Losing a tooth during pregnancy is inevitable. |
| 4 | My baby’s development will draw calcium from my teeth. |
| 5 | Good hygiene is important to minimize oral complications that may arise during pregnancy. |
| 6 | Teeth should be brushed once or more a day during pregnancy. |
| 7 | Going to the dentist during my pregnancy poses a risk to my baby and me. |
| 8 | It is important to see the dentist before, during, and after pregnancy. |
| 9 | It is important to monitor oral health during pregnancy. |
| 10 | I brush my teeth two or more times a day. |
| 11 | I use other oral hygiene methods such as mouthwash, flossing, etc. |
| 12 | I have received information from a dental professional about oral health care during my pregnancy. |
| 13 | I have visited a dentist during my pregnancy. |

| **Clinical variable** | **No night-time toothbrushing (n = 63), mean ± SD** | **Night-time toothbrushing (n = 230), mean ± SD** | **Levene’s p** | **Test used** | **Mean difference** | **95% CI for mean difference** | **p-value** | **Hedges’ g** |
| --- | --- | --- | --- | --- | --- | --- | --- | --- |
| Plaque Index | 1.058 ± 0.310 | 0.471 ± 0.316 | 0.193 | Student’s t-test | 0.587 | 0.499 to 0.675 | <0.001 | 1.859 |
| Gingival Index | 0.828 ± 0.363 | 0.338 ± 0.318 | 0.070 | Student’s t-test | 0.489 | 0.397 to 0.581 | <0.001 | 1.489 |
| Tricolor Index | 1.476 ± 0.309 | 0.768 ± 0.417 | <0.001 | Welch’s t-test | 0.708 | 0.614 to 0.802 | <0.001 | 1.779 |

**Supplementary Table S4.**  **Unadjusted comparison of clinical oral health indicators according to night-time toothbrushing behavior.**

Abbreviations: SD, standard deviation; CI, confidence interval. Homogeneity of variances was assessed using Levene’s test. Student’s t-test was used when equal variances could be assumed, whereas Welch’s t-test was used when the homogeneity assumption was violated. Hedges’ g was used as the standardized effect size.
